# Increasing N-acetylaspartate in the Brain during Postnatal Myelination Does Not Cause the CNS Pathologies of Canavan Disease

**DOI:** 10.3389/fnmol.2017.00161

**Published:** 2017-06-02

**Authors:** Abhilash P. Appu, John R. Moffett, Peethambaran Arun, Sean Moran, Vikram Nambiar, Jishnu K. S. Krishnan, Narayanan Puthillathu, Aryan M. A. Namboodiri

**Affiliations:** Department of Anatomy, Physiology and Genetics and Neuroscience Program, Uniformed Services University of the Health SciencesBethesda, MD, United States

**Keywords:** Acss1, Acss2, aspartoacylase, NAA, NAAG, Nat8L, vacuoles, vacuolization

## Abstract

Canavan disease is caused by mutations in the gene encoding aspartoacylase (ASPA), a deacetylase that catabolizes N-acetylaspartate (NAA). The precise involvement of elevated NAA in the pathogenesis of Canavan disease is an ongoing debate. In the present study, we tested the effects of elevated NAA in the brain during postnatal development. Mice were administered high doses of the hydrophobic methyl ester of NAA (M-NAA) twice daily starting on day 7 after birth. This treatment increased NAA levels in the brain to those observed in the brains of *Nur7* mice, an established model of Canavan disease. We evaluated various serological parameters, oxidative stress, inflammatory and neurodegeneration markers and the results showed that there were no pathological alterations in any measure with increased brain NAA levels. We examined oxidative stress markers, malondialdehyde content (indicator of lipid peroxidation), expression of NADPH oxidase and nuclear translocation of the stress-responsive transcription factor nuclear factor (erythroid-derived 2)-like 2 (NRF-2) in brain. We also examined additional pathological markers by immunohistochemistry and the expression of activated caspase-3 and interleukin-6 by Western blot. None of the markers were increased in the brains of M-NAA treated mice, and no vacuoles were observed in any brain region. These results show that ASPA expression prevents the pathologies associated with excessive NAA concentrations in the brain during postnatal myelination. We hypothesize that the pathogenesis of Canavan disease involves not only disrupted NAA metabolism, but also excessive NAA related signaling processes in oligodendrocytes that have not been fully determined and we discuss some of the potential mechanisms.

## Introduction

Canavan disease is a progressive autosomal genetic leukodystrophy caused by mutations in the gene that codes for the deacetylase enzyme aspartoacylase (ASPA; Matalon et al., 1988). The gene mutations cause loss of ASPA enzyme function which results in an inability to catabolize N-acetylaspartate (NAA), one of the most concentrated metabolites in the human brain. Brain NAA levels vary between healthy adults, and in the same adult with repeated measures over time, with the average whole brain NAA concentration estimated at approximately 12 mM (Rigotti et al., 2011). The physiological function served by such high concentrations occurring in the brain is unknown. The primary documented metabolic effects of mutations in the gene for ASPA in Canavan disease patients are a lack of NAA deacetylation leading to buildup of NAA in the brain (Kvittingen et al., 1986) and greatly increased excretion of NAA in urine (Hagenfeldt et al., 1987). Affected children are developmentally delayed, exhibit hypomyelination and brain vacuolation is progressive with age. A primary question that remains unresolved is whether the etiology involves toxic buildup of NAA or impaired brain metabolism associated with the inability to catabolize NAA, or both. Several hypotheses have been put forward concerning the etiology of Canavan disease, but none has so far been shown conclusively to be the primary pathophysiological mechanism (reviewed in Moffett et al., 2007). The precise involvement of NAA in the pathogenesis of Canavan disease remains controversial primarily because of the uncertainties surrounding the physiological roles of NAA in brain development and function.

The exceptionally high NAA content in mammalian brain was discovered 60 years ago but its physiological importance remains largely unresolved. Only one functional role for NAA has been fairly well established. In the nervous system NAA has been shown to be required for the enzymatic synthesis of the neuronal dipeptide N-acetylaspartylglutamate (NAAG) by NAAG synthetases, and increasing NAA concentrations lead to increased NAAG synthesis (Cangro et al., 1987; Gehl et al., 2004; Arun et al., 2006; Becker et al., 2010; Collard et al., 2010). Additional hypothetical functions for NAA in the nervous system have been proposed. NAA has been proposed to act as an organic osmolyte that removes excess water from neurons by functioning as a molecular water pump (Baslow, 1999, 2002), but this hypothesis has never been directly tested. Microdialysis studies have shown increased NAA in the extracellular fluid in response to hypoosmotic conditions (Taylor et al., 1994, 1995) but other studies of neuronal osmolyte movement in response to osmotic stress have shown NAA to be relatively non-responsive (Tranberg et al., 2007). There is substantial evidence that NAA is a concentrated source of acetate in the brain and contributes acetyl groups for the synthesis of lipids, which in turn are incorporated into myelin (D’Adamo et al., 1968; Burri et al., 1991; Mehta and Namboodiri, 1995; Chakraborty et al., 2001; Madhavarao et al., 2005; Wang et al., 2009), but many questions linking NAA-derived acetate to myelin lipid synthesis remain unanswered.

In addition to the pathologies associated with lack of NAA catabolism and increased concentration of NAA in the brain of Canavan disease patients, a number of studies have suggested that elevated NAA concentrations in the brain may be toxic and lead to cell death. However, the overall evidence linking NAA to cell death is mixed. For example, NAA delivered at very high concentrations to the lateral ventricle (LV) of rats (8 μmol or 1.4 mg NAA in 8 μl of solution, i.e., 1 molar) was found to induce seizures (Akimitsu et al., 2000). Similar results were reported by another laboratory (Kitada et al., 2000). Investigations into the effects of very high concentrations of NAA on cerebral cortex homogenates from 14 day old rats showed that incubation with 40 mM or 80 mM NAA resulted in small but significant reductions in glutathione levels and total antioxidant reactivity (Pederzolli et al., 2007), and reduced catalase activity (Pederzolli et al., 2010). Injection of 8 micromoles of NAA into the cerebral ventricle of 30 day old rats resulted in significantly reduced total radical-trapping potential, catalase and glucose 6-phosphate dehydrogenase activities, as well as increased protein carbonyl content and superoxide dismutase activity (Pederzolli et al., 2009). The authors concluded that high levels of NAA can lead to oxidative damage in the brain. In contrast, other studies have suggested that NAA has anti-inflammatory actions on astrocytes in the central nervous system (CNS; Rael et al., 2004). Recent studies with *Aspa* knockout mice have indicated that an immune system component may be involved in the pathogenesis of Canavan disease (Ahmed et al., 2016).

Orally administered NAA has been found to be non-toxic at relatively high doses. NAA given at acute doses of 2 g/kg or chronic doses of 1 g/kg resulted in no detectable toxic effects in the periphery or CNS (Delaney et al., 2008). At doses of 500 mg/kg/day given to rats every day over the course of two generations, no significant differences were observed in reproductive responses, neurobehavioral tests, ophthalmologic examinations, body weights, feed consumption, or organ weights (Karaman et al., 2011). However, reduced blood urea nitrogen (BUN) was observed in male rats that ingested 500 mg/kg of NAA per day.

Tranberg and Sandberg (2007) used the methyl ester of NAA (M-NAA) to examine NAA toxicity in cell culture. They showed that M-NAA gains access to brain slices in cell culture much more efficiently than non-esterified NAA. Incubation of cultured hippocampal slices with 30 mM NAA did not result in a significant increase in intracellular NAA, whereas incubation with 30 mM M-NAA resulted in a nearly six fold increase in intracellular NAA concentrations with no detectable toxicity (Tranberg and Sandberg, 2007). The authors calculated that this concentration was 2–3 times the normal *in vivo* level, which is similar to the reported level in a mouse model of Canavan disease (Matalon et al., 2000). Similar NAA elevations in the brain have been reported in the tremor rat model of Canavan disease (Kitada et al., 2000). In the current investigation we used orally administered M-NAA to examine the effects of increasing brain concentrations of NAA during postnatal development to determine if high levels of NAA during myelination in the CNS lead to any of the overt pathologies observed in Canavan disease. This is the first study to examine the effects of using M-NAA to raise brain NAA levels *in vivo* during the course of postnatal brain development and myelination.

## Materials and Methods

M-NAA was obtained from Bachem, Switzerland, methanol was from Merck, AG50W X8 resin was from BioRad and paraformaldehyde, gavage needles, MDA assay kits and Tween 20 were from Sigma Aldrich. Total reactive oxygen species (ROS) kits were from Enzo Life Sciences. NuPAGE 10% Bis-Ris gels were from Invitrogen. All antibodies used in Western blotting were from Cell Signaling.

### Animals

Male and female C57BL/6 breeder mice were obtained from Taconic Biosciences (Hudson, NY, USA) and the 7 day old and 30 day old mice used in this study were bred in the Uniformed Services University of the Health Sciences animal facility. Once weaned (approximately 23 days old), the mice were given continuous access to food and water. *Nur7* breeder mice (mouse model of Canavan disease) were provided by Dr. Brian Popko, University of Chicago and *Aspa* deficient pups were bred from heterozygous (*Aspa*^+/Nur7^) females and homozygous (*Aspa*^Nur7/Nur7^) males that were 2–5 months old. All procedures were performed in accordance with guidelines of the National Institutes of Health and the animal protocol was approved by the Uniformed Services University of the Health Sciences Institutional Animal Care and Use Committee.

### Dose Response Study and LC-MS/MS Analysis of NAA

For the dose response study, 12 30-day old wild type mice were grouped randomly into four groups, three animals in each group including control (no treatment) and treatment groups administered M-NAA at doses of 1 g/kg, 3 g/kg, and 10 g/kg body weight. Animals were euthanized by pentobarbital injection 2 h after treatment and brains were collected and processed for liquid chromatography tandem mass spectroscopy (LC-MS/MS) analysis. Brain tissue was extracted with methanol, centrifuged and the supernatant collected. The supernatants were lyophilized and re-dissolved in distilled water and subjected to cation exchange column chromatography. The column was washed with water to collect the unbound NAA and the washes were lyophilized and re-dissolved in 2% formic acid. LC-MS/MS analyses were carried out on an Agilent 1200 series HPLC system with a Higgins Analytical TARGA C18 3 mm 2.1 × 100 mm reverse phase column (Nest Group Inc., Southborough, MA, USA) maintained at 20°C. The mobile phase was composed of solvent A: 0.2% formic acid in water and solvent B: 0.2% formic acid in acetonitrile; the samples were injected (5 μl) and eluted with isocratic flow (200 μl/min) of 100% solvent A for 5 min, followed by a 3 min isocratic period of 50% A/50% B to flush the column and an 8 min isocratic period of 100% A to re-equilibrate the column prior to the next injection. The HPLC output flow was directed into the TurboV electrospray ionization (ESI) source of a Q-Trap 4000 mass spectrometer (AB Sciex, Framingham, MA, USA). LC-MS/MS analyses were performed in positive mode with the ion source temperature of 350°C, a spray voltage of 5.5 kV and a declustering potential of 35 V. Multiple reaction monitoring (MRM) of NAA was performed on the transition *m/z* 176.1 Da → 134.1 Da; an enhanced product ion (EPI) scan was also run in order to confirm the identity of NAA by observing the other fragment ions (158.1, 130.0, 116.0 and 87.9 Da) in approximately the same intensity pattern as observed for an authentic NAA standard. A calibration curve was constructed using nine concentrations of NAA in water from 5.7 nM to 103 nM (1.0–18 ng/ml); this concentration range is appropriate for the 1000× fold dilution of the samples following the cation exchange column step in the sample processing before LC-MS/MS.

### Toxicity Studies

Developmental toxicity studies were initiated in 7 day old wild type mouse pups, which were assigned randomly to three experimental groups (control, methanol and M-NAA) with nine mice in each group. Out of these mice, one subgroup consisting of six mice per group was used for toxicity studies (Western blot, ROS and serology) and another subgroup, consisting of three mice per group, was used for histopathology. M-NAA and methanol were dissolved in distilled water and given by oral gavage to mice in the respective groups once in the morning and once in the evening starting from day 7 after birth and continued for 30 days. Based on the LC-MS/MS results we chose an M-NAA dose of 5 g/kg, which was expected to raise brain NAA levels by 2.5–3 fold over controls. The methanol group (MeOH) received methanol at a dose of 0.84 g/kg (the molar equivalent to M-NAA). Methanol was used as a control because hydrolysis of M-NAA yields methanol plus NAA. We did not measure brain NAA levels at the end of the 30 day treatment. At the end of the experimental period, animals were anesthetized with pentobarbital and blood was collected by cardiac puncture. Brains were extracted and rapidly frozen on dry ice within 1–2 min after the mice were sacrificed. ROS measurements were done the same day as the tissue was collected, whereas the tissues for MDA assays were stored frozen for several days at −80°C until analyzed. Serology was performed by VRL laboratories, Rockville, MD, USA.

### Total Antioxidant, ROS and MDA Determinations

ROS were detected with the Total ROS Detection Kit as per the manufacturer’s instructions (ENZ- 51010; Enzo Life Sciences). MDA was estimated by using lipid peroxidation MDA assay kit from Sigma Aldrich according to the manufacturer’s instructions.

### Western Blot Analysis

SDS-PAGE and Western blotting of brain proteins was done as previously described (Hershfield et al., 2006). Tissue homogenates were prepared with a T-PER™ Tissue Protein Extraction Reagent (Thermofisher). Homogenates were centrifuged (16,000 *g*, 10 min, 4°C) and the supernatants were collected and protein concentrations determined with a BCA protein assay kit (Pierce). Nuclear proteins were obtained from tissue homogenates using a Nuclear Extraction Kit (Abcam; ab113474) according to the manufacturer’s instructions.

Aliquots containing 50 μg of protein per sample were loaded onto gels. Following electrophoresis, samples were transferred to Immobilon-P PVDF membranes, blocked with albumin and incubated overnight with respective antibody produced from rabbit (1:1000 dilution) at 4°C. Membranes were washed in PBS and then incubated for 1 h with horseradish peroxidase-conjugated goat anti-rabbit secondary antibody at a dilution of 1:1000. Membranes were washed and developed with Pierce ECL Western Blotting Substrate (Thermofisher) and acquired and quantified the intensity of band by using ChemiDoc touch imaging system (Bio-Rad). Expression of β-Actin was used as control measure for the ratio for protein expression in the cytoplasm and histone H3 for expression in the nucleus.

Antibodies used for Western blotting included: from Cell Signaling: acetyl-p53 (Lys382) antibody #2525, cleaved caspase-3 (Asp175) antibody #9661, IL-6 (D5W4V) XP® rabbit monoclonal (mouse specific) #12912, nuclear factor (erythroid-derived 2)-like 2 (NRF2) (D1Z9C) XP® rabbit monoclonal #12721, β-actin (13E5) rabbit monoclonal #4970, and from Abcam; NOX2/gp91phox antibody [EPR6991] (ab129068). All antibodies were used at a dilution of 1:1000.

### Histology

For histology and immunohistochemistry, nine 1-month old animals were used (three per group). Animals were anesthetized and perfused transcardially with approximately 30 ml of 10% neutral buffered formalin. Brains were postfixed overnight in the same solution. Formalin-fixed brains were saturated with 30% sucrose, blocked, mounted on chucks with OCT compound, frozen with compressed gas and sectioned in the coronal plane on a cryostat at −20°C (20 μm section thickness). The sections were mounted on slides and stained with cresyl violet acetate or hematoxylin for standard histopathology and evaluation of potential vacuolation.

### Peroxidase Immunohistochemistry

Avidin-biotin complex/peroxidase immunohistochemistry was done as previously described (Ariyannur et al., 2010a; Arun et al., 2010; Moffett et al., 2011). The sections were processed free-floating. Endogenous peroxidase activity was blocked by washing freshly cut sections in PBS and incubating them in a 50:50 mixture of methanol and water containing 1% H_2_O_2_ for 30 min. The sections were blocked against non-specific antibody binding by incubation in PBS containing 2% NGS and 0.1% sodium azide.

Antibodies used in the study included polyclonal antibodies to glial fibrillary acidic protein (GFAP; Abcam #ab7260, 1:8000 dilution), purified polyclonal Aspa antibodies (dilution 1:20,000) (Madhavarao et al., 2004), polyclonal antibodies against claudin-11, also known as oligodendrocyte specific protein (Abcam #ab53041, dilution 1:4000), polyclonal antibodies to parvalbumin (Abcam #ab11427, dilution 1:10,000) and purified polyclonal antibodies to acyl-CoA short chain synthase family member 2 (Acss2, dilution 1:4000) which have been previously described (Ariyannur et al., 2010a). The Acss2 and Aspa antibodies were purified by pre-adsorption against tissues from a gene null animal (*Acss2*^−/−^ and *Nur7*) as previously described (Moffett et al., 2011). Briefly, the crude sera were diluted 1:100 in 2% normal goat serum in PBS plus 0.1% sodium azide and incubated overnight with a large excess of formalin-fixed tissue sections (20 μm thickness; brain, liver, kidney and spleen) from *Acss2* knockout mice or *Nur7* mice. The sections were removed from the solution, and the process repeated two more times to generate antibody preparations that were highly specific for Acss2 and Aspa.

Brain tissue sections were incubated for 16–24 h (up to 48 h for claudin-11) with the primary antibodies at room temperature with constant rotary agitation. Bound antibodies were visualized by the avidin-biotin complex method with horseradish peroxidase as the enzyme marker (Vectastain Elite; Vector Labs, Burlingame, CA, USA). The sections were incubated with the biotinylated secondary antibody and avidin-peroxidase complex solutions for 90 min each. The biotinylated secondary antibody was diluted in PBS plus 2% NGS and the ABC reagent in PBS plus 0.5% bovine serum albumin (BSA), both without azide. After final washing, the sections were developed with a Ni and Co enhanced diaminobenzidene chromogen (Pierce Chemical Co, Rockford, IL, USA).

Stained tissue sections were transferred to treated slides (Superfrost plus, Fischer Scientific). Slides were dried at 45°C for 10–15 min, dehydrated in an ethanol series, cleared in xylene and sealed with cover glasses using a xylene-based adhesive. Images were acquired on an Olympus BX51 microscope with an Olympus DP71 digital camera, Additional software was used to correct for background illumination levels (Image Pro Plus; Media Cybernetics, Silver Spring, MD, USA) and to adjust white balance and brightness with PC imaging software (Photoshop; Adobe Systems, San Jose, CA, USA). Some higher magnification images were generated using extended depth of field, where multiple images (6–11) are taken at different focal planes within the 20 μm thick tissue slices, and combined using software (Image Pro Plus ver. 7.1, Media Cybernetics, Rockville, MD, USA).

### Statistical Analysis

The results were analyzed using GraphPad Prism-7 and SPSS/PC+, Version 22 (SPSS Inc., Chicago, IL, USA). One way ANOVA was used for comparison among the group. Duncan’s *post hoc* multiple comparison tests of significant differences among groups were determined, and *p* ≤ 0.05 was considered to be significant.

## Results

Following 30 days of M-NAA treatment of the young, wild type mice, we examined a number of potential etiological factors in the vacuolization characteristic of Canavan disease ranging from ROS production to immune activation, as well as various pathological consequences including vacuole formation, GFAP immunoreactivity and lipid peroxidation. We did not examine a number of other factors, including behavioral differences, or possible long term changes in health or longevity. Nonetheless, we did not observe any pathological changes that were characteristic of Canavan disease.

### Dose Dependence Study

**Figure 1:**
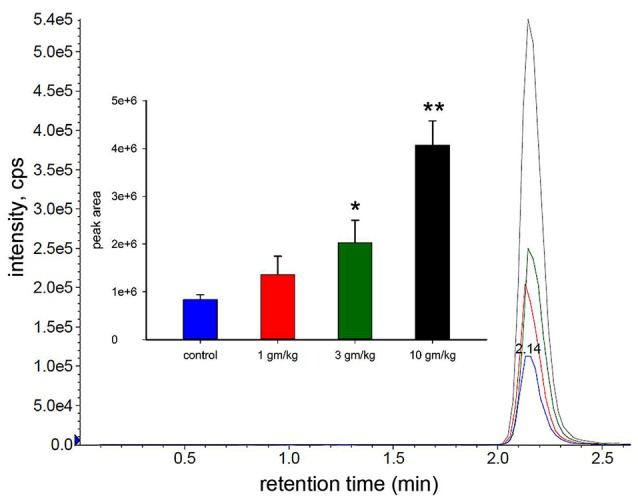
Liquid chromatography-mass spectrometry (LC-MS/MS) analysis of N-acetylaspartate (NAA) in mouse brain after acute dosing with methyl ester of NAA (M-NAA). Typical LC-MS/MS scan results for the four groups (blue = control, red = 1 g/kg, green = 3 g/kg, black = 10 g/kg). Graph insert shows the peak areas for each group (means ± SD, *n* = 3 per group). Brain NAA levels in the 3 g/kg group were approximately 2.4 times those of controls which is similar to the levels found in aspartoacylase (ASPA) deficient mice (*p = 0.01, **p = 0.0004, Student’s *t*-test, two tailed, two sample equal variance).

In the dose dependence study we treated 30 day old wild type mice with different doses of M-NAA (1, 3 and 10 g/kg) to find out the availability of NAA in the brain using LC-MS/MS analysis. The results showed that the NAA levels were significantly increased in the 3 and 10 g/kg treated groups in comparison with controls (Figure 1). An approximate 2.4 fold increase in brain NAA was observed with an M-NAA dose of 3 g/kg, whereas an approximate 5-fold increase was observed in the 10 g/kg group in comparison with control. In an Aspa knockout Canavan mouse model the level of NAA in the brain is approximately 2.3 times that in measured in age-matched wild type mice (Matalon et al., 2000). Therefore, we selected 5 g/kg as optimum dose for further detailed study.

### Toxicity Markers in Serum

Standard toxicity markers were analyzed in serum (see Tables 1–3). Results showed that the activities of most parameters were significantly increased in the MeOH treated group in comparison with controls, indicating mild peripheral toxicity in the MeOH treated rats. No significant alterations were observed in the M-NAA treated group in comparison with controls with the exception of BUN, which was reduced in the M-NAA group as compared with controls. Similar results on BUN levels have been reported previously when NAA was administered at high doses in the diet (Karaman et al., 2011). Creatinine levels were unchanged in the M-NAA group so the BUN/creatinine ratio was also reduced significantly relative to controls.

**Table 1:** Activities of toxicity markers in serum.

Group	Albumin (g/dl)	ALT (U/L)	ALP (U/L)	AST (U/L)	CPK (U/L)	LDH (U/L)
Control	2.76 ± 0.23	18.0 ± 6.3	111 ± 16.1	150 ± 45.1	513 ± 253	571 ± 164.5
MeOH	3.35 ± 0.43*	77.0 ± 24.7*	213 ± 108.4*	286 ± 115.3*	781 ± 252	1032 ± 373.4*
M-NAA	2.85 ± 0.45	20.1 ± 4.4	140 ± 31.6	132 ± 79.8	310 ± 324	508 ± 156.2

Values are expressed as means ± SD with six mice per group. No changes in serum enzyme markers of toxicity were observed with M-NAA treatment, whereas significant toxicity was observed with equimolar methanol treatment. Abbreviations: ALT, alanine aminotransferase; ALP, alkaline phosphatases; AST, aspartate aminotransferase; CPK, creatine phosphokinase; LDH, lactate dehydrogenase. *Denotes significant difference relative to controls (*p* ≤ 0.05, t test; two tailed, equal variance).

**Table 2:** Levels of glucose, triglycerides and cholesterol in serum.

Group	Glucose (mg/dl)	Triglycerides (mg/dl)	Cholesterol (mg/dl)
Control	207.7 ± 20.6	97.3 ± 13.1	130.0 ± 15.7
MeOH	222.0 ± 19.3	78.5 ± 8.2*	131.3 ± 16.8
M-NAA	236.2 ± 21.5	84.3 ± 23.6	125.3 ± 15.5

Values are expressed as means ± SD of six mice per group. *Denotes significant difference relative to controls (*p* ≤ 0.05, t test; 2 tailed, equal variance).

**Table 3:** Levels of creatinine, BUN and BUN/creatine ratio in serum.

Group	Creatinine (mg/dl)	BUN (mg/dl)	BUN/Creatinine ratio
Control	0.18 ± 0.03	21.0 ± 2.89	119.8 ± 26.5
MeOH	0.19 ± 0.04	21.0 ± 4.43	116.9 ± 38.9
M-NAA	0.19 ± 0.02	15.3 ± 3.88*	80.4 ± 15.3*

BUN and the BUN/creatine ratio were significantly lower in the M-NAA treated group. Values are expressed as means ± SD of six mice per group. *Denotes significant difference relative to controls (p ≤ 0.05, t test; two tailed, equal variance).

### Total Antioxidants, ROS and MDA Levels in Brain

**Figure 2:**
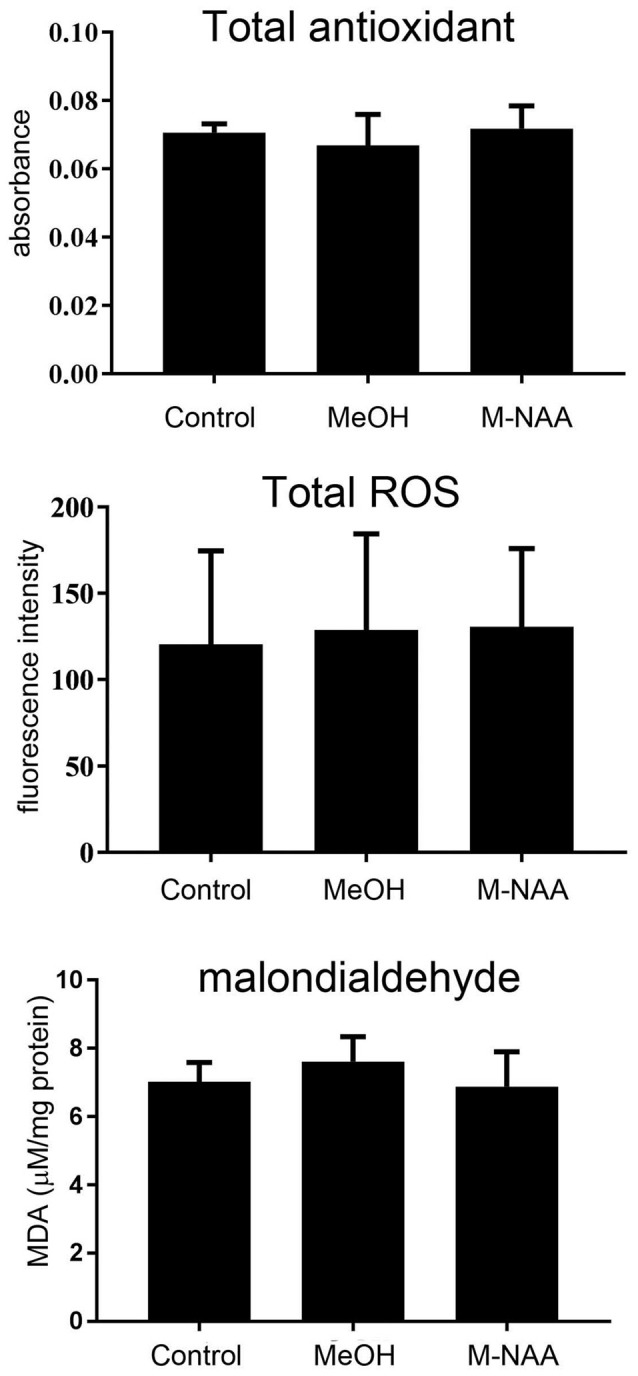
Total antioxidants, reactive oxygen species (ROS) and MDA levels in brain; Values are expressed as means ± SD (*n* = six mice per group). No significant differences were observed in any measure among the three groups.

For evaluation of oxidative stress and lipid peroxidation, we analyzed the total antioxidants, ROS and MDA in brain. The results showed that oxidative stress and lipid peroxidation markers were not altered in the MeOH and M-NAA groups in comparison with controls (Figure 2).

### Protein Expression of Interleukin-6, NADPH Oxidase and Cleaved-Caspase-3

**Figure 3:**
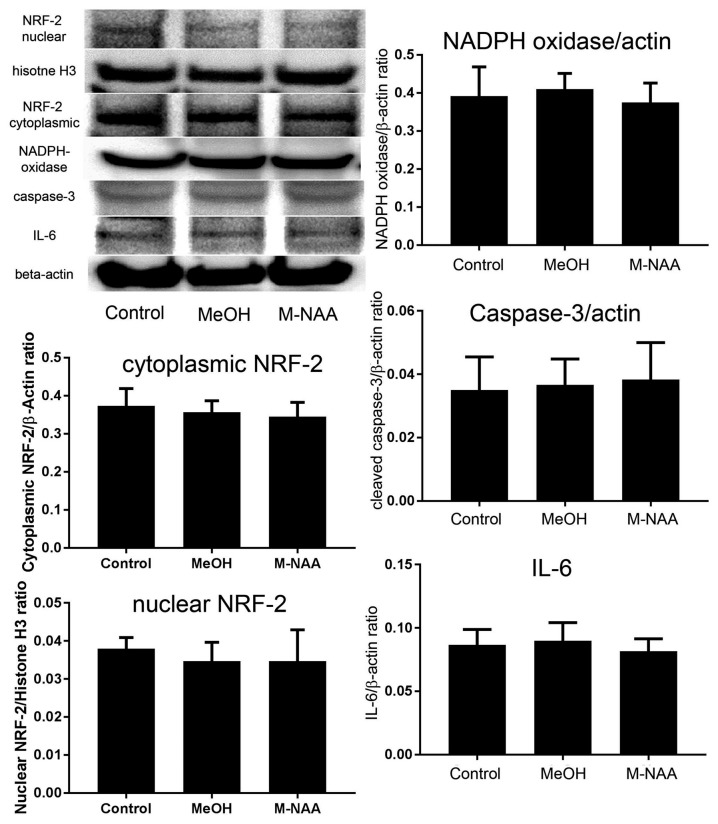
Western blot analyses of inflammation markers. Western blots were used to assess expression levels of several markers of inflammation and stress responses including interleukin-6, cleaved caspase-3 and NADPH oxidase. No significant differences were observed among the groups. We also evaluated expression of the cytoprotective transcription factor nuclear factor (erythroid-derived 2)-like 2 (NRF-2) in both cytoplasmic and nuclear fractions and no translocation of NRF-2 to the nucleus was observed. Values indicate means ± SD with *n* = 6 per group.

Western blot analyses indicated that protein expression for the inflammation marker interleukin-6 and the apoptosis marker cleaved caspase-3 were not altered in the MeOH or M-NAA groups in comparison with controls (Figure 3). Further, the expression of the oxidative stress marker NADPH oxidase was unchanged in either treatment group.

### Translocation of NRF-2

The nuclear translocation of the antioxidant enzyme-inducing transcription factor NRF-2 was evaluated in both cytoplasmic and nuclear fractions. Results showed that the expression of NRF-2 was not altered in either the cytoplasmic or nuclear fractions of MeOH and M-NAA groups in comparison with control (Figure 3). This finding indicates that the cells were not subjected to sufficient damage to initiate protective responses.

### Histology and Immunohistochemistry

**Figure 4:**
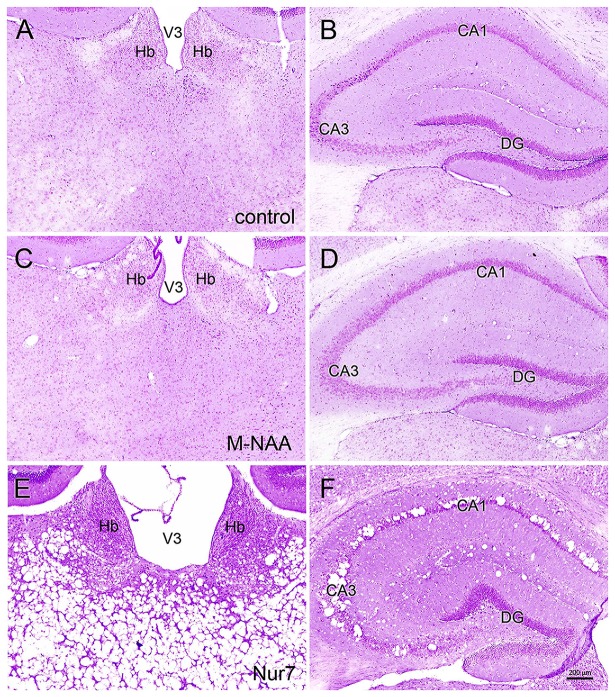
Histopathology. Representative hematoxylin staining in the dorsal thalamus and hippocampus of control **(A,B)**, M-NAA treated **(C,D)** and *Nur7* mice **(E,F)**. No vacuoles or other histopathological changes were observed after 30 day treatment with either M-NAA (5 g/kg twice per day) or methanol (0.84 mg/kg twice per day; data not shown). Extensive vacuolization was observed in many brain regions of *Nur7* mice, including the thalamus and hippocampus **(E,F)**. Abbreviations: CA1 and CA3, CA1 and CA3 pyramidal cell layers of hippocampus; DG, dentate gyrus of hippocampus; Hb, habenula; V3, third ventricle. Micron bar = 200 μm.

**Figure 5:**
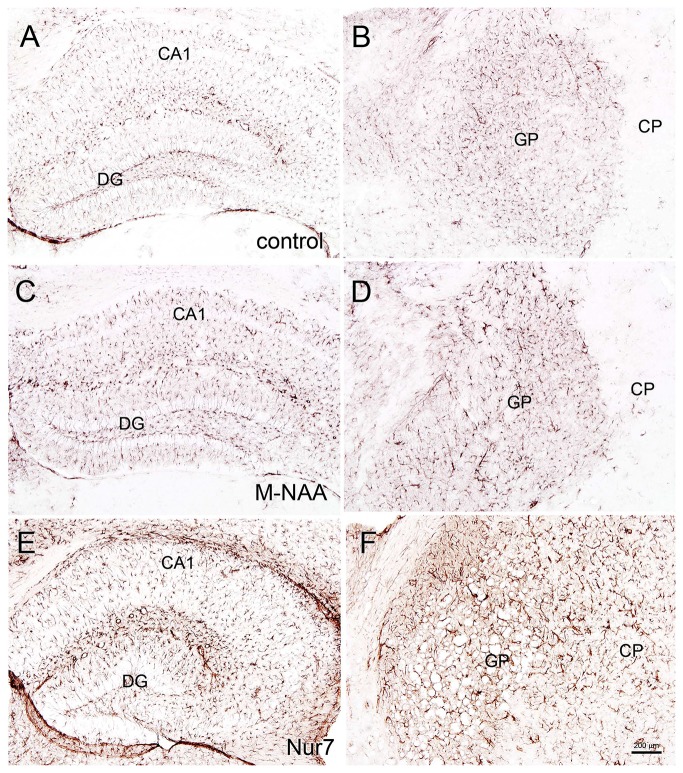
Glial fibrillary acidic protein (GFAP) immunohistochemistry. Representative sections stained for GFAP in the hippocampus and globus pallidus (GP) of control **(A,B)** and treated mice. No changes in immunoreactivity were observed after 30 days of treatment with either M-NAA (**C,D**; 5 g/kg twice per day) or methanol (0.84 mg/kg twice per day; data not shown). GFAP immunoreactivity was substantially increased in *Nur7* mice **(E,F)**. Abbreviations: CA1, CA1 pyramidal cell layer of hippocampus; DG, dentate gyrus of hippocampus; GP, globus pallidus. Micron bar = 200 μm.

**Figure 6:**
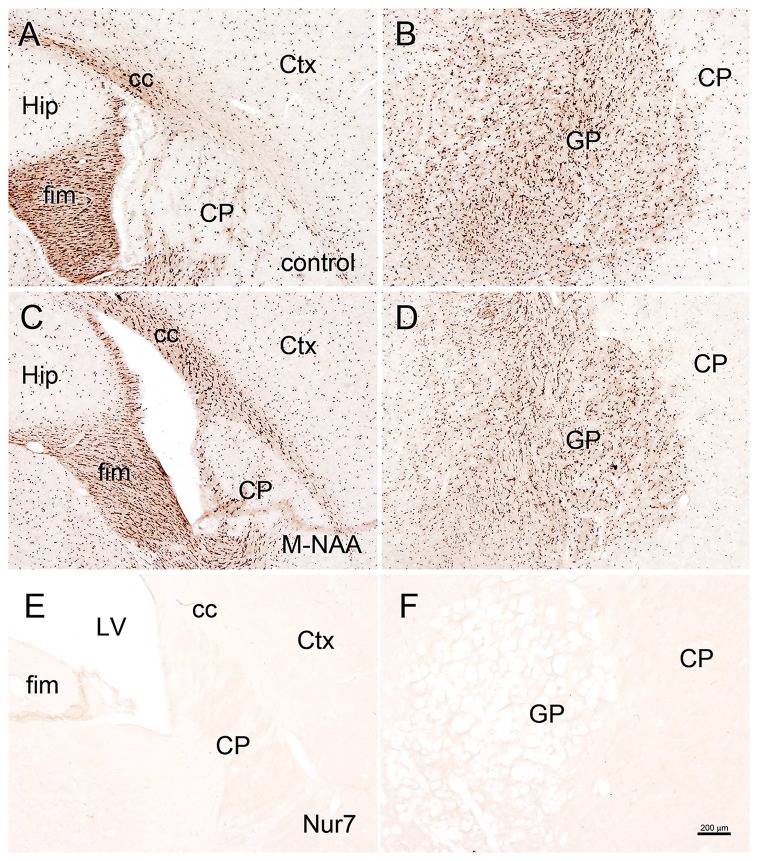
ASPA immunohistochemistry. Aspa immunoreactivity was very strong in oligodendrocytes throughout the brain in untreated wild type mice **(A,B)**. Minor animal to animal staining variability was observed with antibodies to Aspa, but no consistent changes in immunoreactive intensity were observed after 30 days of treatment with either M-NAA **(C,D)** or methanol (data not shown) treatment. Aspa immunoreactivity was completely absent in *Nur7* mice **(E,F)**. Abbreviations: cc, corpus callosum; CP, caudate-putamen; Ctx, cortex; GP, globus pallidus; fim, fimbria; Hip, hippocampus; LV, lateral ventricle. Micron bar = 200 μm.

**Figure 7:**
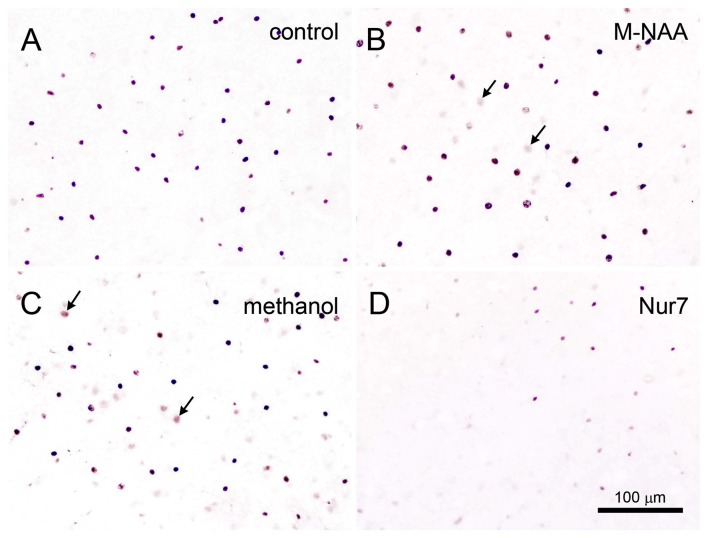
Acyl-CoA short chain synthetase family member 2 (Acss2) immunohistochemistry. Acss2 immunoreactivity was sparse in control mice **(A)**, occurring almost exclusively in scattered cell nuclei throughout the brain. Immunoreactivity was slightly increased (arrows) in some cell nuclei in the M-NAA **(B)** and methanol groups **(C)**. In contrast, Acss2 immunoreactivity was drastically reduced in the untreated *Nur7* mouse brain **(D)**. All images are from layers II–V of neocortex. Micron bar = 100 μm.

Vacuolization is extensive in the brains of *Nur7* mice as shown by standard histological staining with cresyl violet acetate or hematoxylin. The localization of vacuoles in *Nur7* mice was very consistent between animals. Vacuoles were present in specific neuronal groups, with the most extensive damage in forebrain occurring in the central thalamus (Figure 4). Severe vacuolization was also present in the globus pallidus (GP), whereas the caudate nucleus was almost completely spared from vacuoles. Most major fiber pathways were devoid of vacuoles. The consistent and specific vacuolization pattern in *Nur7* mouse brains indicates that some specific cell types are susceptible to high internal NAA levels or defective NAA catabolism, and others are not. Standard histological staining revealed no pathological CNS changes in either the M-NAA or methanol treated groups and no vacuoles were observed in any area (Figure 4). Immunohistochemistry for GFAP showed no increase in immunoreactivity in either treated group (Figure 5). Aspa expression in the brain was also unchanged by either treatment (Figure 6). Acss2 expression was relatively sparse in control mouse brain, occurring predominantly in scattered cell nuclei (Figure 7). Immunoreactivity for Acss2 was similar or slightly increased in the M-NAA and methanol groups, but was dramatically reduced in *Nur7* mouse brain sections relative to controls. The reduction in staining was very noticeable in regions with extensive vacuolization in *Nur7* brains. In the forebrain, staining for Acss2 in the *Nur7* mice was near normal levels only in the superficial layers of neocortex, where no vacuolization was observed.

**Figure 8:**
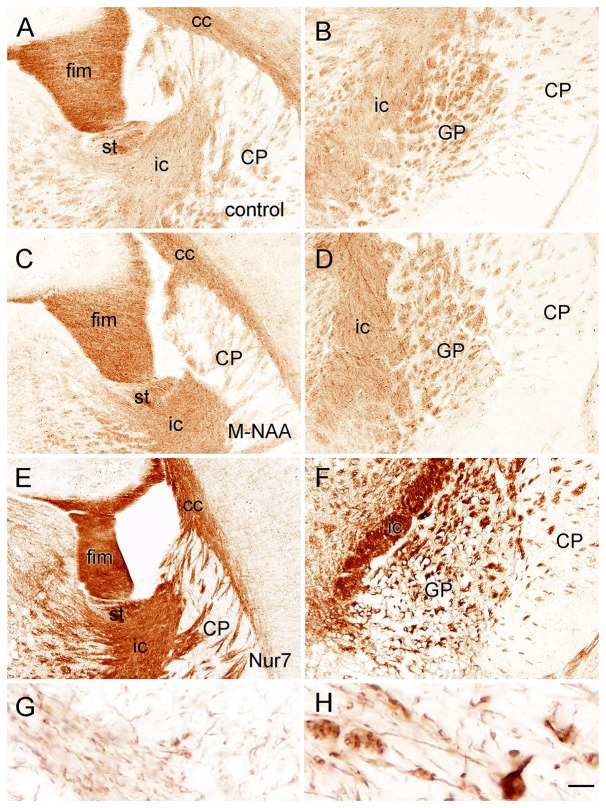
Immunoreactivity for claudin-11 (also known as oligodendrocyte specific protein) in the forebrain. Staining for claudin-11 was moderate to strong in fiber pathways in control mice **(A,B)**, and there was no change in immunoreactivity with M-NAA treatment **(C,D)**. In contrast, Claudin-11 immunoreactivity was highly elevated in all fiber pathways in untreated *Nur7* mice **(E,F)**. Major fiber pathways such as the fimbria of the hippocampus and internal capsule were reduced in thickness in *Nur7* mice, suggesting axonal and oligodendrocyte loss. Oligodendrocyte processes were enlarged with swollen segments in *Nur7* mice **(H)** as compared with controls **(G)**. Abbreviations: cc, corpus callosum; CP, caudate/putamen; fim, fimbria; ic, internal capsule; GP, globus pallidus; st, stria terminalis. Micron bar = 200 μm in **(A–F)**, 10 μm in **(G,H)**.

Claudin-11 is a tight junction protein associated with myelin that may act to limit water and solute movement between myelin layers (Chow et al., 2005; Gow and Devaux, 2008; Denninger et al., 2015). In the current study immunoreactivity for claudin-11 was unaffected by treatment with methanol or M-NAA. In contrast, claudin-11 immunoreactivity was highly elevated in the brains of *Nur7* mice (Figure 8). Claudin-11 positive oligodendrocyte processes appeared swollen in *Nur7* mice and fiber pathways were stained much more intensely than in control or M-NAA treated mice. M-NAA treatment did not result in increased claudin-11 expression, as was the case with *Nur7* mice (Figure 8).

**Figure 9:**
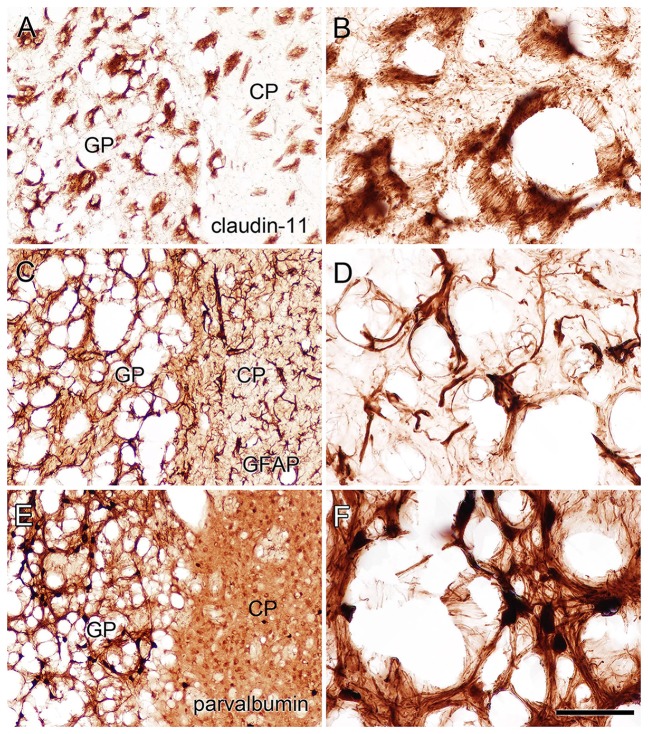
Staining for claudin-11 (oligodendrocytes; **A,B**), GFAP (astrocytes; **C,D**) and parvalbumin (neurons; **E,F**) showed that all three cell types remained in heavily vacuolated areas such as the GP. Higher magnification images in **(B,D,F)** are from heavily vacuolated areas in the GP (extended depth of field). Micron bar = 200 μm **(A,C,E)**, 50 μm **(B,D,F)**.

Immunohistochemistry for GFAP (astrocytes), claudin-11 (oligodendrocytes) and parvalbumin (neurons) showed that all three cell types were present in the heavily vacuolated areas such as the GP (Figure 9). This indicates that despite cell loss leading to vacuole formation, some neurons, oligodendrocytes and astrocytes may be more resistant than others and remain in the most heavily damaged brain regions.

## Discussion

The enzymes involved in NAA synthesis and breakdown are aspartate N-acetyltransferase (Asp-NAT; Truckenmiller et al., 1985) and Aspa respectively (Kaul et al., 1991). The gene encoding Asp-NAT has been identified as* NAT8L* (Ariyannur et al., 2010b; Wiame et al., 2010). Mutations in the *ASPA* gene result in Canavan disease, which leads to hypomyelination, severe developmental delay, brain vacuolization and early death (Hagenfeldt et al., 1987; Matalon et al., 1988). The only reported case of loss of *NAT8L* in a human patient showed significant developmental delay, microcephaly and seizures, but with less overt pathology than is seen in severe Canavan disease patients (Burlina et al., 2006; Wiame et al., 2010). However, not all *Aspa* mutations result in severe cases of Canavan disease, and some cases are relatively mild (Tacke et al., 2005; Janson et al., 2006). Reports have indicated that mutations resulting in lower conformational or thermal enzyme stability lead to the more severe cases of the disorder (Zano et al., 2013; Wijayasinghe et al., 2014). A recent study has indicated that even low levels of residual ASPA activity associated with certain *ASPA* mutations result in milder forms of Canavan disease (Mendes et al., 2017).

Brain vacuolization occurs in humans with Canavan disease (Adachi et al., 1973), as well as the *Aspa*-deficient rodent models (Figure 4; Surendran et al., 2005; Traka et al., 2008; Mersmann et al., 2011). Canavan disease shares many features with other genetic leukodystrophies that adversely affect mitochondrial function (Szalardy et al., 2016) or impair lipid synthesis (Rolyan et al., 2015) including CNS vacuolization. The pathophysiological cause of the vacuole formation in Canavan disease has become more enigmatic as additional data has been forthcoming, and in particular, gene knockout studies have provided unexpected results. Knockout of the gene for the NAA biosynthetic enzyme (gene, *Nat8L*) in mice does not result in any severe pathology or vacuolization, but does result in behavioral differences even in the heterozygous (*Nat8L* +/−) condition (Ariyannur et al., 2013; Toriumi et al., 2015). These results show that the loss of *Nat8L* results in a much milder pathophysiology than occurs with a loss of *Aspa*. Moreover, the simultaneous loss of both *Aspa* and *Nat8L* prevents brain vacuolization and hypomyelination, strongly suggesting that a buildup of NAA in the brain is a major cause of the pathologies observed in *ASPA* deficient humans and animal models (Guo et al., 2015). In a comprehensive study of mice that are deficient in both *Nat8L* and *Aspa* it was confirmed that the dual knockout mice did not exhibit hypomyelination or brain vacuolization (Maier et al., 2015). However, these double knockout mice had shortened lifespans that were closer in longevity to those of *Aspa* deficient mice than wild type mice. Further, the double knockout mice had altered myelin structure and lipid composition. In mice that were heterozygous for *Nat8L* (+/−) as well as *Aspa* null (nur7/nur7), brain NAA levels were about 80% of those seen in control mice and brain vacuolization was present, but less severe than in mice that were lacking *Aspa* only (*Nur7/Nur7*). These findings strongly implicate increased NAA in the absence of Aspa in the vacuolization observed in Canavan disease. It can be concluded that the CNS pathology results from the presence of NAA in the absence of Aspa, i.e., intracellular NAA must be degraded in oligodendrocytes to prevent the pathology. This assessment fits with the data in the current study where greatly increased NAA during brain development did not result in vacuolization (Figure 4) or increased GFAP immunoreactivity (Figure 5) most likely due to the presence of active Aspa enzyme in the brain (Figure 6). Increased brain NAA is not pathogenic during development as long as sufficient Aspa is present and functional. Postnatal restoration of *Aspa* gene function in mouse models of Canavan disease have shown clearly that functional Aspa expression during postnatal myelination prevents vacuolization and normalizes brain development (Ahmed et al., 2013, 2016; Francis et al., 2016; Gessler et al., 2017).

**Figure 10:**
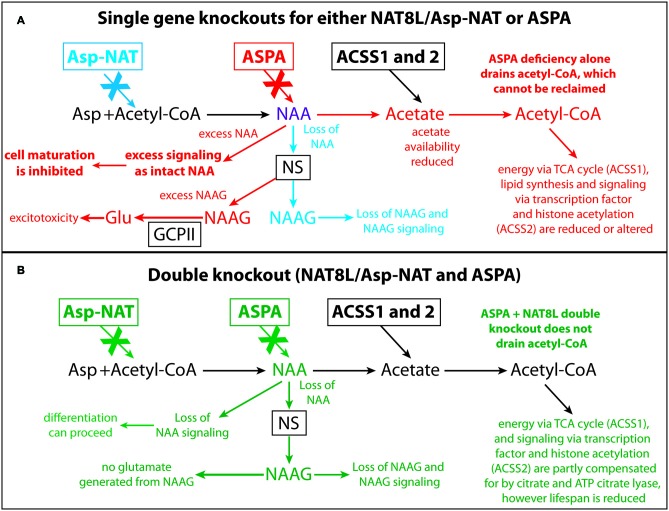
Schematic depicting the effects of *Nat8L* and *Aspa* gene knockouts and associated pathophysiological changes. Single gene deletion effects for *Nat8L*/Asp-NAT (blue) and *Aspa* (red) are shown in **(A)**, whereas changes associated with the double knockout mice (green) are shown in **(B)**. Only the effects associated with the single *Aspa* gene knockout (red) result in brain vacuolization. Two major differences between the single ASPA knockout mice and the dual *Nat8L*+*Aspa* knockout are that in the double knockout mice acetyl-CoA is not depleted and trapped as NAA, and excess NAA signaling or other potentially toxic actions from NAA are not possible. Further, no NAAG signaling occurs in the dual knockout mice, whereas excess NAAG is present in the single *Aspa* deficient mice. Acss1 and Acss2 convert acetate, including acetate derived from Aspa-mediated deacetylation of NAA, into acetyl-CoA for energy derivation, lipid synthesis and protein acetylation reactions. In Aspa deficiency alone, acetyl-CoA is removed from the system, and cannot be reclaimed. This affects downstream utilization of acetate by Acss1 and Acss2. Acetyl-CoA is not depleted in the *Nat8L*+*Aspa* double knockout mice because no acetyl-CoA is used to synthesize NAA. Abbreviations: Asp, aspartate; Asp-NAT, aspartate N-acetyltransferase (Nat8L); ASPA, aspartoacylase; Acss1 and 2, acyl-CoA short chain synthetase 1 and 2; Glu, glutamate; GCPII, glutamate carboxypeptidase-2; NAAG, N-acetylaspartylglutamate; NS, NAAG synthetase.

Gene knockout studies showing vacuolization and other pathologies of Canavan disease to be absent when both *Aspa* and *Nat8L* (Asp-NAT) are ablated provide strong evidence that the buildup of NAA in oligodendrocytes and/or neurons is deleterious (Guo et al., 2015; Maier et al., 2015; Toriumi et al., 2015; Ahmed et al., 2016; Sohn et al., 2017). However, most studies on the effects of high NAA concentrations on cells have failed to show serious toxic effects that could account for the severe vacuolization observed in Aspa deficiency. In the present study we showed that substantially elevated NAA in the brain during postnatal development (over 2.3-fold; Figure 1) does not lead to any of the pathological features of Canavan disease, and does not increase any stress or inflammation markers in the brain (Figures 2, 3). Further, based on the very high levels of NAA observed in the urine of Canavan patients (Al-Dirbashi et al., 2007), it is apparent that the brain has effective clearance mechanisms for removing excess NAA. Another potential factor in the pathophysiology of ASPA deficiency is that the increase in NAA concentration results in an increase in NAAG levels (Burlina et al., 1999). For example, increased NAAG catabolism via glutamate carboxypeptidases could result in the production of excess glutamate, which could potentially lead to excitotoxicity (Figure 10). It has been shown that oligodendrocytes respond to NAA and NAAG application, but in the same study it was demonstrated that 1 mM NAAG applied to oligodendrocytes for 6 h did not result in an increase in cell death (Kolodziejczyk et al., 2009). NAAG levels were not measured in the present study, but it is likely that brain levels in the M-NAA treated mice were elevated due to the increased NAA concentrations, which have been shown to increase NAAG synthesis (Cangro et al., 1987; Gehl et al., 2004; Arun et al., 2006).

It is noteworthy that serum markers were altered in the MeOH treated group indicating peripheral toxicity (Tables 1, 2), whereas in the M-NAA treated group only BUN and the BUN/creatinine ratio were changed (Table 3). Creatinine levels were unchanged in the M-NAA group indicating normal kidney function. Reduced BUN and BUN/creatinine ratios in the range observed in the M-NAA treated group are not indicative of kidney or liver dysfunction, and similar results on BUN have been reported in a previous study of male rats fed 500 mg/kg NAA per day for 90 days (Karaman et al., 2011). The kidney has higher Aspa expression and activity levels than the brain (Hershfield et al., 2006; Sommer and Sass, 2012), and it is possible that NAA catabolism in the kidneys facilitates or enhances urea excretion by an unknown mechanism. NAA has been proposed to facilitate nitrogen removal from the brain (Moffett et al., 2007), but potential roles for NAA in nitrogen balance have not been investigated in any tissue.

Despite all of the information provided by recent gene knockout studies, it is still difficult to discern the specific mechanisms responsible for brain vacuolization resulting from Aspa deficiency. The lack of consensus on the pathophysiological mechanism stems from the lack of understanding of the key functions served by NAA synthesis and breakdown in the brain. Several potential mechanisms have been proposed over the last several decades, but the more recent data still does not strongly favor one mechanism over the other. The range of possible NAA-related mechanisms involved in the extensive brain vacuolization in Canavan disease, which are not mutually exclusive, include: (1) NAA has detrimental pro-oxidant (Pederzolli et al., 2007, 2009, 2010) or osmotic effects in neurons or oligodendrocytes (Baslow, 1999, 2002, 2017); (2) energy metabolism is compromised in neurons and/or oligodendrocytes due to impaired NAA catabolism (Francis et al., 2012, 2014, 2016); (3) a lack of NAA catabolism results in altered acetyl-coenzyme A metabolism or availability for lipid synthesis and protein acetylation (D’Adamo et al., 1968; Burri et al., 1991; Madhavarao et al., 2005; Moffett et al., 2007, 2013; Ariyannur et al., 2010b); and (4) NAA has important but as yet unknown functions that are disrupted by a lack of catabolism in oligodendrocytes.

Two key differences between the young, M-NAA treated wild type mice in the current study and developing Aspa deficient mice are that NAA can continue to be broken down in oligodendrocytes in the M-NAA treated wild type mice, and the elevated levels of NAA in the brains of M-NAA treated mice are probably spread more uniformly throughout the brain rather than being concentrated in any particular cell type. Other differences that may be pertinent include the direction and mechanism of NAA transport. In one case NAA originates in neurons and is passed to oligodendrocytes, presumably by dicarboxylate transporters, in the other case an NAA precursor is delivered via the bloodstream, and diffuses throughout the brain where it is converted to NAA in all cell types. It remains to be determined whether the lack of intracellular NAA catabolism or the resulting increased concentration of NAA leads to oxidative damage, osmotic stress, energy failure or excessive signaling or some combination of these mechanisms in oligodendrocytes. Recent discoveries on NAA metabolism in brown fat tissue support the role of NAA in energy derivation and lipid turnover (Pessentheiner et al., 2013). Subsequent studies showed that NAA in brown adipocytes was involved in gene regulation through changes in histone methylation and acetylation (Prokesch et al., 2016). Inhibition of Aspa expression in these cells led to reduced acetyl-CoA levels and histone H3 acetylation indicating that NAA-derived acetate was involved in providing acetyl-CoA for epigenetic regulation of chromatin remodeling.

In order for NAA-derived acetate to be used for energy derivation, lipid synthesis or protein acetylation reactions, it must first be converted to acetyl-CoA. The enzymes involved in converting free acetate to acetyl-CoA are known as ACSS and three forms are currently known (family members 1–3) (Yoshimura et al., 2017). These enzymes expend ATP to generate acetyl-CoA from acetate and coenzyme A. ACSS1 and ACSS3 are mitochondrial matrix enzymes involved in the oxidation of acetate and propionate respectively for energy derivation, whereas Acss2 is a nuclear-cytoplasmic enzyme involved in lipid synthesis and protein acetylation reactions. Acss2 expression in the adult rat brain occurs predominantly in the nuclei of neurons, astrocytes and oligodendrocytes (Ariyannur et al., 2010a). In the current study M-NAA and methanol treatment slightly increased Acss2 immunoreactivity in the brain, but the increase involved a minor increase in some cell nuclei (arrows in Figures 7B,C). This is in contrast to Acss2 immunoreactivity in the *Nur7* mouse brain, which was reduced to extremely low levels (Figure 7D). Acss2 immunoreactivity in the *Nur7* mice was virtually absent in many brain regions with extensive vacuolization, such as central thalamus. These findings suggest that lack of Aspa in combination with high NAA levels inhibit Acss2 expression. Recently it has been shown in brown adipocytes that *Aspa* knockdown or addition of exogenous NAA led to decreased expression of Acss2, and the effects of the two treatments were additive (Prokesch et al., 2016). In fact, a number of genes were found to be significantly downregulated with low Aspa activity in conjunction with high NAA including *Acss1*, *Nat8L*, ATP citrate lyase and adipose triglyceride lipase. The connection between low Aspa expression in conjunction with high NAA levels and reduced gene expression is uncertain, but we hypothesize that NAA, as an intact molecule, has significant regulatory effects on gene transcription.

The results from a number of studies support the conclusion that Canavan disease is a disorder of oligodendrocyte maturation. Investigations have implicated the lack of NAA catabolism in blunting or preventing oligodendrocyte maturation, resulting in a loss of both oligodendrocytes and neurons (Kumar et al., 2006, 2009). We have previously observed that NAA promotes growth and inhibits differentiation in primary tumor derived glioma stem-like cells and oligodendrocyte progenitor cells (Long et al., 2013). NAA at a concentration of 100 μM inhibited cAMP-mediated differentiation of these cell types suggesting that in some cell types NAA is involved in facilitating or maintaining an undifferentiated state through second messenger pathways. Subsequent studies on NAA metabolism in cancer cells provide evidence that NAA is involved in energy derivation from glutamine (glutaminolysis) (Wynn et al., 2016) and biomass accumulation (Zand et al., 2016). Further, *NAT8L* silencing was found to inhibit cancer cell proliferation in a number of different cancer cell types (Lou et al., 2015; Weindl et al., 2016). Our investigations into the expression of Aspa (Hershfield et al., 2006; Moffett et al., 2011) and Acss2 (Ariyannur et al., 2010a; Arun et al., 2010) led us to hypothesize that NAA-derived acetate is involved in the epigenetic regulation of gene transcription via protein acetylation reactions (Moffett et al., 2013). Recently NAA has been shown to regulate histone acetylation and methylation in oligodendrocytes. Oligodendrocytes responded to low micromolar concentrations of NAA by altering histone methylation patterns, and reducing histone H3 acetylation (Singhal et al., 2017). These findings indicate that NAA is involved directly (via intact NAA) and/or indirectly (via acetyl-CoA generation) in regulating chromatin remodeling and gene transcription.

In the current study immunoreactivity for claudin-11, a tight junction protein associated with myelin, was highly upregulated in the brains of *Nur7* mice but was not increased by M-NAA treatment in wild type mice (Figure 8). The increase in claudin-11 expression in the brains of *Nur7* mice was associated with increased diameter of the stained oligodendrocyte processes, which also exhibited swollen, disorganized segments as compared with controls (Figures 8G,H). In regions with extensive vacuolization, such as the GP, immunoreactivity for claudin-11, GFAP and parvalbumin in the remaining tissue indicated that oligodendrocytes, astrocytes and neurons persisted despite the severe pathology (Figure 9). The morphology was abnormal in all three cells types in the remaining tissue surrounding the vacuoles (Figures 9B,D,F).

Based on the available evidence we hypothesize that in addition to roles in lipid synthesis, energy derivation and regulatory actions via protein acetylation, NAA has unidentified signaling functions as an intact molecule and that high intracellular concentrations of NAA suppress cellular differentiation and favor proliferation in certain cell types (Figure 10). In this view Aspa serves two roles, one to regulate the intracellular concentration of NAA to maintain proper levels for signaling as an intact molecule, and a second role to generate acetyl-CoA in specific subcellular compartments for use in lipid synthesis, energy derivation and protein acetylation. This would explain the results of *Aspa* knockout studies where loss of Aspa enzyme activity results in high intracellular NAA levels in oligodendrocytes and failure to mature and properly myelinate axons. Ablation of NAA biosynthesis alone or in conjunction with *Aspa* knockout would result in low intracellular NAA concentrations in oligodendrocytes, and allow oligodendrocyte maturation to proceed. It is likely that the loss of acetyl-CoA in oligodendrocytes that results from a lack of NAA-derived acetate in *Nat8L* and *Nat8L/Aspa* knockout mice is mostly compensated for by increased citrate production in oligodendrocyte mitochondria via citrate synthase. However, myelination is affected as can be seen in the reduced sphingolipid synthesis and altered myelin structure in the *Nat8L/Aspa* deficient mice (Singhal et al., 2017). The degree to which acetyl-CoA production from citrate via ATP citrate lyase can compensate for the loss of NAA-derived acetate is uncertain, but in most cell types the major proportion of extramitochondrial acetyl-CoA is derived from citrate. The singular loss of *Aspa* function would trap some proportion of acetate as NAA, which could not be further metabolized. If NAA supplied by neurons is an important source of acetyl-CoA in oligodendrocytes during brain development, then it is possible that certain populations of oligodendrocytes are more susceptible to the loss of this source of acetyl-CoA than others, resulting in cell death and vacuolization.

The generation of a conditional *Aspa* knockout mouse line may help answer some of the questions concerning the roles that NAA plays in the pathogenesis of Canavan disease. If *Aspa* silencing after oligodendrocyte maturation and postnatal myelination are complete fails to result in brain vacuolization, then the vacuolization is not simply due to NAA toxicity or osmotic stress; it is a disorder of brain development. The formation of vacuoles in *Aspa* deficient animals coincides with oligodendrocyte maturation and myelination. This suggests that there is a specific and limited time frame where NAA excess and/or loss of NAA catabolism can interfere with brain development. We hypothesize that NAA has as yet unidentified signaling functions as an intact molecule in oligodendrocytes whereby high NAA levels act to inhibit oligodendrocyte maturation. This could occur, for example, if NAA acts as a selective histone deacetylase (HDAC) inhibitor or has actions on specific transcription factors. In this scenario, high NAA levels in developing oligodendrocytes, as well as in certain cancer cells, promotes proliferation and blunts maturation (Long et al., 2013). This would explain how oligodendrocytes can complete the maturation process in *Nat8L/Aspa* double knockout mice, because no NAA is synthesized and no NAA signaling occurs. Aspa regulates the intracellular NAA concentration in oligodendrocytes, and in *Aspa* deficiency NAA levels in oligodendrocytes cannot be regulated, intracellular levels rise substantially, and the maturation process cannot be completed normally. In double KO mice NAA levels in oligodendrocytes remain low and maturation can proceed, but due to lack of NAA and NAA-derived acetyl-CoA, myelin pathologies result and lifespan is substantially reduced. Based on the available evidence we hypothesize that intact NAA has specific regulatory actions on transcription factors or other cell cycle control elements in oligodendrocytes and other cell types (e.g., brown adipocytes) that are important for proper cellular maturation.

## Conclusion

NAA buildup in neurons and oligodendrocytes is clearly involved in the pathogenesis of Canavan disease as shown by double (*Aspa* and *Nat8L*) knockout studies. The expression of Aspa in the brain is very strong in oligodendrocytes, including both their cytoplasm and nucleus. It remains to be determined whether Aspa expression in oligodendrocytes acts to remove excess NAA, prevent excess NAAG synthesis, control NAA-related signaling or provide acetate for the generation of acetyl-CoA for energy, lipid synthesis and signaling activity through protein acetylation reactions. It seems likely that some combination of these factors contribute to the vacuole formation in Canavan disease (Figure 10). We hypothesize that higher intracellular levels of NAA act to blunt cellular differentiation in some cell types or in certain physiological contexts, and this may have implications for cancer as well as Canavan disease. The vacuolization pattern in *Nur7* mice is highly stereotypical, occurring in specific neuronal groups and some fiber pathways while sparing other neuronal groups and the majority of fiber pathways. This specific pattern is not indicative of a general toxicity, or a global osmotic dysregulation, but rather suggests that some cell types are susceptible to high NAA and/or NAAG levels, whereas others are not. If intact NAA or excess NAAG are only damaging to specific populations of developing oligodendrocytes or neurons, then it is likely that one or both of these compounds have important signaling or regulatory functions during brain development in the susceptible cells. Additional studies on protein acetylation and methylation in oligodendrocytes in response to increased NAA, in combination with Aspa inhibition, may provide some of the answers to these longstanding questions on the etiology of Canavan disease and may also shed light on important connections between altered NAA metabolism and cancer.

## Author Contributions

APA, JRM, JKSK and AMAN conceived and designed the project. APA, JRM, PA, SM, VN, JKSK and NP conducted the research. APA, JRM and AMAN composed the figures and wrote the manuscript.

## Conflict of Interest Statement

The authors declare that the research was conducted in the absence of any commercial or financial relationships that could be construed as a potential conflict of interest.
